# Intergeneric hybridization of two stickleback species leads to introgression of membrane-associated genes and invasive TE expansion

**DOI:** 10.3389/fgene.2022.863547

**Published:** 2022-08-25

**Authors:** Artem Nedoluzhko, Fedor Sharko, Svetlana Tsygankova, Eugenia Boulygina, Natalia Slobodova, Anton Teslyuk, Jorge Galindo-Villegas, Sergey Rastorguev

**Affiliations:** ^1^ Paleogenomics Laboratory, European University at Saint Petersburg, Saint Petersburg, Russia; ^2^ Limited Liability Company ELGENE, Moscow, Russia; ^3^ Laboratory of Vertebrate Genomics and Epigenomics, Research Center of Biotechnology of the Russian Academy of Sciences, Moscow, Russia; ^4^ Laboratory of Bioinformatics and Big Data Analysis, Kurchatov Center for Genomic Research, National Research Center “Kurchatov Institute”, Moscow, Russia; ^5^ Laboratory of Eukaryotic Genomics, Kurchatov Center for Genomic Research, National Research Center “Kurchatov Institute”, Moscow, Russia; ^6^ National Research Center “Kurchatov Institute”, Moscow, Russia; ^7^ Faculty of Biosciences and Aquaculture, Nord University, Bodø, Norway

**Keywords:** *Gasterosteus aculeatus*, hybridization, introgression, membrane-associated genes, *Pungitius pungitius*, stickleback, transposable elements

## Abstract

Interspecific hybridization has occurred relatively frequently during the evolution of vertebrates. This process usually abolishes reproductive isolation between the parental species. Moreover, it results in the exchange of genetic material and can lead to hybridogenic speciation. Hybridization between species has predominately been observed at the interspecific level, whereas intergeneric hybridization is rarer. Here, using whole-genome sequencing analysis, we describe clear and reliable signals of intergeneric introgression between the three-spined stickleback (*Gasterosteus aculeatus*) and its distant mostly freshwater relative the nine-spined stickleback (*Pungitius pungitius*) that inhabit northwestern Russia. Through comparative analysis, we demonstrate that such introgression phenomena apparently take place in the moderate-salinity White Sea basin, although it is not detected in Japanese sea stickleback populations. Bioinformatical analysis of the sites influenced by introgression showed that they are located near transposable elements, whereas those in protein-coding sequences are mostly found in membrane-associated and alternative splicing-related genes.

## 1 Introduction

The process of hybridization between two different species, which is known as interspecific hybridization, is one of the most crucial events occurring during the evolutionary process for all kingdoms of life. Previously, interspecific hybridization has long been considered as a rare process, resulting in the destruction of reproductive isolation between species (Borkin and Litvinchuk, 2013). Moreover, this point of view was strengthened by the concept of postzygotic isolation (PSI), which postulates that allele incompatibility between different species makes distant hybridization more difficult (Dobzhansky, 1940). Recent studies, however, have shown that evolutionarily successful interspecific hybridization is widely common in nature. At least 25% of plant and more than 10% of animal species (including vertebrates) show traces of hybridization in their genomes (Mallet, 2005).

It seems that, despite PSI, successful introgressive hybridization plays an important role in the evolutionary process it is involved in vertebrate speciation (Abbott et al., 2013; Mallet et al., 2016), by helping to acquire new traits (Chiba, 2005; Pereira et al., 2014a; Nichols et al., 2015), increase genetic variation, and conquer new habitats (Lee, 2002; Dlugosch and Parker, 2008; Prentis et al., 2008; Yoshida et al., 2016). Traces of introgressive hybridization between species can, nowadays, be precisely detected using high-throughput sequencing, which allows us to conduct studies of this phenomenon on an interspecific and even intergeneric level (Jombart and Ahmed, 2011; Payseur and Rieseberg, 2016; Runemark et al., 2019; Taylor and Larson, 2019).

Interspecific hybridization is also quite common among fish species (Cui et al., 2013; Wallis et al., 2017). The development of whole-genome sequencing (WGS) and bioinformatic methods allowed to show that interspecific hybridization between fish species is a relatively common source for the acquisition of new traits (Ford et al., 2015; Souissi et al., 2018; Runemark et al., 2019).

The three-spined stickleback (*Gasterosteus aculeatus*) and its quite distant mostly freshwater relative the nine-spined stickleback (*Pungitius pungitius*), both in the order Gasterosteiformes, are widely used for ecological, evolutionary, and functional biology studies (Merila, 2013; Rastorguev et al., 2016; Artemov et al., 2017; Rastorguev et al., 2018; Yoshida et al., 2019). The nine-spined stickleback is mostly a freshwater species, whereas the three-spined stickleback has marine and freshwater ecological forms which are significantly distinct because of divergent selective biotic and abiotic pressures that influence their natural selection. Interestingly, the marine form of three-spined stickleback can survive in freshwater conditions for extended periods and often spawns in freshwater streams and rivers flowing into a sea. Previously, it has been shown that the freshwater three-spined stickleback has genomic sites, also known as “divergence islands,” which increase its capacity for adaptation to freshwater conditions (Jones et al., 2012; Terekhanova et al., 2014; Terekhanova et al., 2019).

The possibility of hybridization between the marine and freshwater forms of three-spined stickleback has been demonstrated, and the resulting hybrid offspring have expanded ecological potential compared with their parental forms (Taylor et al., 2006; Lucek et al., 2010; Rudman and Schluter, 2016). Moreover, interspecific hybridization between two Gasterosteus species—*G. aculeatus* and *G. nipponicus*—apparently facilitates freshwater habitat colonization by the three-spined stickleback (Yoshida et al., 2016). The possibility of interspecific hybridization between *Pungitius* species has also been observed. The nine-spined stickleback (*P. pungitius*) can form fertile interspecific hybrids with either the Sakhalin stickleback (*P. tymensis*) or the Ukrainian stickleback (*P. platygaster*), with the ability to induce viable backcrosses (Kobayashi, 1959).

Genomic divergence between three- and nine-spined sticklebacks is very high. The divergence time of these species is estimated to be within 25.5–28.8 M years (Varadharajan et al., 2019). Notably, the chromosome number of the nine-spined stickleback coincides with that of the three-spined stickleback, but differs from much more closely related species such as the four-spined stickleback (*Apeltes quadracus*) and the brook stickleback (*Culaea inconstans*), of which the karyotypes have 23 pairs of chromosomes (Urton et al., 2011). However, it should be noted that nine-spined stickleback chromosomes have 70 chromosome arms, whereas three-spined stickleback chromosomes have only 58 (Varadharajan et al., 2019). This may result in difficulties in chromosome segregation during meiosis and to fertility reduction of the hybrids. Nevertheless, the artificial hybrids between three- and nine-spined sticklebacks that grow to maturity can reproduce themselves (Hart, 2003), which implies their existence is likely a result of occasional hybridization between these two species in natural conditions.

In our previous study based on transposable elements (TEs) and restriction site-associated DNA sequencing (RAD-Seq) analyses, we described, for the first time, the existence of genomic introgression between three- and nine-spined stickleback species (Nedoluzhko et al., 2021). RAD-Seq analysis is applicable for the identification of hybridization traces in admixture genomes, but it is much more complex to reveal the genomic location of these introgressed loci. At the same time, the analysis of genomic localization of these loci could shed light on the molecular mechanisms of interspecific introgression between these distant species. TE-analysis and RAD-sequencing of eight specimens from the White Sea stickleback population revealed one that produced a clearly noticeable sign of admixture. This finding prompted us to suspect the possibility of introgression between these two sympatric stickleback species residing in northwestern Russia (Nedoluzhko et al., 2021).

In the present study, using whole-genome sequencing of three- and nine-spined stickleback specimens from the White Sea basin, we clearly demonstrate a moderate level of introgression between three- and nine-spined sticklebacks in White Sea populations of these species. We show that introgressed loci in the nine-spined stickleback genome are most frequently located in genes encoding membrane-related proteins and alternative splicing-associated factors.

## 2 Materials and methods

### 2.1 Sampling

Three- and the nine-spined sticklebacks were collected from the White Sea area near the Chkalovsky village in the Republic of Karelia, Russia. Two three-spined stickleback specimens were collected in the coastal zone of the White Sea and in the estuary (near the tidal zone) of the Chkalovsky stream, which drains into the White Sea. Nine-spined stickleback specimens were collected in the estuary (near the tidal zone) and in the headwaters of the Chkalovsky stream as well as in a quarry located near Chkalovsky village (Table 1). One nine-spined stickleback specimen (Pun1) which presented a reduction in the number of spines and showed introgression traces in its genome based on transposable and RAD-sequencing analyses (Nedoluzhko et al., 2021) was also used to conduct whole genome sequencing.

**TABLE 1:** Nomenclature and sampling location of the three- and nine-spined stickleback (*G. aculeatus and P. pungitus,* respectively) specimens used in this study.

Specimen	Library name	Species	Sampling location (^a^)	Sampling date	Sampling positions (GPS
Gas1	LibB3−PP	*G. aculeatus*	CS, E	08/07/2014	66.298057, 33.400795
Gas2	Lib311	*G. aculeatus*	WS	08/07/2014	66.298404, 33.320415
Pun1	LibB2−P9M	*P. pungitius*	CS, E	07/07/2014	66.298057, 33.400795
Pun2	LibChu82	*P. pungitius*	CS, E	07/07/2014	66.298057, 33.400795
Pun3	LibChu83	*P. pungitius*	CS, E	07/07/2014	66.298057, 33.400795
Pun4	LibD1	*P. pungitius*	CS, upstream	07/07/2014	66.296781, 33.398263
Pun5	LibChM42	*P. pungitius*	CS, upstream	07/07/2014	66.296781, 33.398263
Pun6	LibChM72	*P. pungitius*	CS, upstream	07/07/2014	66.296781, 33.398263
Pun7	LibB9−1	*P. pungitius*	Quarry near, CV	06/07/2014	66.298542, 33.344362
Pun8	LibK3	*P. pungitius*	Quarry near, CV	06/07/2014	66.298542, 33.344362
Pun9	LibK7	*P. pungitius*	Quarry near, CV	06/07/2014	66.298542, 33.344362

a All specimens were collected in the Republic of Karelia, Russia. Chakalovsky stream (CS), Chakalovsky village (CV), Estuary (E), White Sea (WS).

### 2.2 DNA extraction and sequencing

Genomic DNA was extracted from fin clips using the phenol–chloroform extraction method. The digestion was performed with proteinase K at 60°C for 4–5 h (Sambrook et al., 2006). DNA quantity was determined with a Qubit 2.0 (Thermo Fisher Scientific, United States). DNA integrity was assessed by 1% agarose gel electrophoresis.

DNA libraries were constructed using the NEBNext Ultra II DNA Library Prep Kit for Illumina (NEB, United States). Amplified DNA libraries were quantified using a high-sensitivity chip on a 2100 Bioanalyzer instrument (Agilent Technologies, United States). The S2 flow cell of the Illumina NovaSeq 6000 genome analyzer (Illumina, United States) was used for DNA library sequencing of 150 bp paired end reads.

### 2.3 Illumina read mapping, SNP calling, genotyping analysis

Raw Illumina reads from three-spined stickleback libraries were mapped to the three-spined stickleback reference genome (Ensembl accession: BROAD S1) and Illumina reads from nine-spined stickleback libraries were mapped to the nine-spined stickleback reference genome (NCBI accession: PRJEB33823) (Varadharajan et al., 2019) using the Bowtie2 software package (v. 2.3.4.1) with the*–very-sensitive* parameter (Langmead and Salzberg, 2012). Mapping statistics is presented in Table 3.

The mapped data, in SAM format, were converted to BAM format, and were then sorted and indexed using the SAMtools package (v 0.1.19) (Li et al., 2009). SNP calling was conducted with BCFtools software (v 1.9) (Li et al., 2009) with a minimum base quality of 30 (*--min-BQ* parameter) and with depth coverage information for each SNP loci as an INFO tag to output in the VCF file (*--annotate DP* parameter). The VCF files, which were obtained using BCFtools, were loaded into the R statistical environment (www.r-project.org) using the vcfR package (Knaus and Grunwald, 2017). Then, we filtered out the SNP loci according to their coverage. Only the high-quality SNPs (with *p*-value < 0.05; coverage > 10×) were used in the subsequent analysis. We converted data in VCF format into the genlight format of the adegenet R package (Jombart and Ahmed, 2011) and used the StAMPP R package to calculate Nei’s distances (Pembleton et al., 2013). Clustering based on Nei’s distance matrix was conducted using the nj () funtion in the ape R package (Paradis and Schliep, 2019).

### 2.4 ABBA-BABA test

The genome of European bass (*Dicentrarchus labrax*, diclab1, PRJEB5099) was used as an outgroup for the ABBA-BABA (or D-statistic) test. D-statistic test is used to detect introgression between samples (e.g., species). This test requires three samples (e.g., species) and an outgroup. Positive test results indicate the presence of introgression. The sequencing data of each stickleback specimen were mapped to the *D. labrax* reference genome using the Bowtie2 software package (v. 2.3.4.1) with the*–very-sensitive-local* parameter (Langmead and Salzberg, 2012). Mapping statistics is presented in Table 3. The mapped data, in SAM format, were converted to BAM format, and were then sorted and indexed using the SAMtools package (v 0.1.19) (Li et al., 2009). The resulting BAM files and ANGSD software suite (Korneliussen et al., 2014) were used for the ABBA-BABA test (where diclab1 genome FASTA file represents the outgroup). This type of analysis provides a simple and powerful test for a deviation from strict bifurcating evolutionary history by considering only those loci for which the genotypes are known in all the specimens studied.

### 2.5 Mobile elements analysis

De novo identification and analysis of transposable elements in both reference genomes were conducted as previously described (Arkhipova, 2017; Sharko et al., 2019) using the REPET package (Flutre et al., 2011), which combines three mutually complementary repeat identification tools (RECON, GROUPER, and PILER). The outputs were subjected to additional classification with the RepeatClassifier tool from the RepeatMasker package (www.repeatmasker.org), which was also used to build the corresponding TE landscape divergence plots. TEs described in three- and nine-spined stickleback genomes were then used in the method described in Section 2.7: “Correlation of introgressed genomic intervals to genes and transposons.”

We used BAM files of three- and nine-spined stickleback from Japanese populations to identify transposable elements that were specific only for three-spined stickleback. We collected TE loci which were completely covered in the three-spined stickleback genome, though not completely covered in the nine-spined stickleback genome (depth of coverage >100×; breadth of coverage <80%). The number of such “three-spined” transposons was estimated for each specimen. The breadth of coverage was determined using the coverage function after converting BAM files to the BED format using the bedtools package (Quinlan and Hall, 2010).

### 2.6 “Alien” read rate estimation

The main problem in the specimen comparison was significant genomic differences between the three- and nine-spined stickleback species*.* In this study, we could not compare the genomic differences of our specimens based on a single reference genome.

The Illumina reads of each specimen were mapped to the three-spined stickleback (Ensembl accession: BROAD S1) and to the nine-spined stickleback (NCBI accession: PRJEB33823) reference genomes using the Bowtie2 software package (v. 2.3.4.1) with the*–very-sensitive* parameter (Langmead and Salzberg, 2012).

**FIGURE 1:**
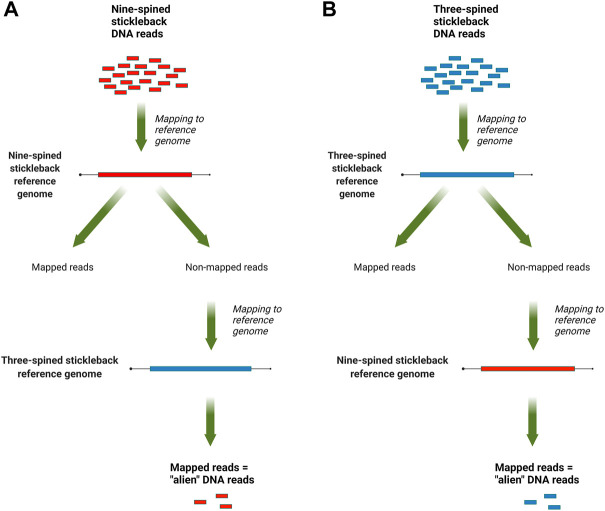
An overview of the “alien” read rate estimation pipeline: **(A)** for nine-stickleback sequencing data; **(B)** for three-spined stickleback sequencing data.

The mapped data, in SAM format, were analyzed with a custom Perl script that counted reads that mapped to the reference genome of other species but not mapped to the species’ own reference genome. For three-spined stickleback specimens, we counted reads which mapped to the nine-spined stickleback genome but not to the three-spined stickleback genome, and vice versa for the nine-spined stickleback reads. An overview of this pipeline is presented in Figure 1. To normalize the results, the counts were scaled by the total number of reads, which were produced for each DNA library.

### 2.7 Identification and analysis of introgressed loci

We used the sppIDer package to define loci of the three-spined stickleback that were transferred to the admixed specimen (the source of introgression) (Langdon et al., 2018). We created a joined reference genomic sequence for three- and nine-spined sticklebacks according to the software algorithm, and all of the DNA reads were then mapped against this. The fragments of the three-spined stickleback genome covered by the reads from the nine-spined stickleback specimen were expected to be a source of introgression loci.

However, to provide further support to our date, we mapped DNA paired-end reads to three- and nine-spined stickleback reference genomes but considering the relationship between forward and reverse tags to determine the introgression target regions in the nine-spined stickleback genome.

This detailed analysis was necessary to link the three-spined stickleback genome’s loci considered as the source of introgressed loci with the nine-spined stickleback genome representing the target of the introgressed loci.

Note that, we determined only those paired-end reads in which one of tag of the pair-end read exclusively mapped to the three-spined stickleback genome, whereas the second type of tag exclusively mapped to the nine-spined stickleback genome. The coordinates of these second type of tags were considered to represent introgressive loci. The resulting coordinates of introgression target loci were converted to BED format and merged into a single BED file using the bedtools package (Quinlan and Hall, 2010).

### 2.8 Correlation of introgressed genomic intervals to genes and transposons

Genomic intervals corresponding to introgressed loci of each specimen were defined with sppIDer software (Langdon et al., 2018). Then, we removed genomic positions with low coverage (threshold >10) and merged adjacent nucleotides into extended intervals using the bedtools merge command (Quinlan and Hall, 2010).

Mobile element intervals were taken as described above in Section 2.4, “Mobile elements analysis”. The genomic intervals corresponding to three-spined stickleback genes were obtained from the Ensembl database FTP server (ftp://ftp.ensembl.org/pub/release-100/gtf/gasterosteus_aculeatus/) (Howe et al., 2020), and gene annotation for the nine-spined stickleback genome was obtained from the Figshare service https://figshare.com/collections/The_assembly_and_annotation_of_stickleback/4548146 (Varadharajan et al., 2019).

Genomic intervals were loaded from BED files to the GenomeRanges R package (Lawrence et al., 2013) and GenometriCorr R package (Favorov et al., 2012) was used to assess for intersections and the proximity of each type of interval. Pairwise analyses were conducted for introgressed loci versus TEs, as well as introgressed loci versus genes.

### 2.9 Introgressed gene set analyses

Sequences of introgressed loci were obtained as described above in Section 2.6: “Introgressed loci identification and analysis.” The “alien” reads were *de novo* assembled into “alien” contigs using SPAdes (v.3.10) software with the--rna parameter (Bankevich et al., 2012). These contigs were aligned to the three-spined stickleback gene database using the BLAST + package (Camacho et al., 2009). three-spined stickleback genes were obtained from the Ensemble database (Howe et al., 2020) using the BioMart data mining tool (Kinsella et al., 2011).

The BLAST results table was parsed using a custom Perl script to define the highest scored genes. The top 500 stickleback gene names were converted to official gene names with the BioMart tool (Kinsella et al., 2011). Only 205 of the 500 selected genes had universal names, and these genes were analyzed using the DAVID 6.8 functional annotation web service (Huang et al., 2009).

## 3 Results

The total number of reads generated for eleven three- and nine-spined stickleback specimens varied from 54,187,021 to 129,103,432 per DNA library. Furthermore, DNA reads of two stickleback specimens from Japanese populations were obtained from the Sequence Read Archive (SRA). SRA accession numbers are presented in Table 2. Genome mapping efficiency is presented in Table 3.

**TABLE 2:** Illumina-generated reads used in this study and Sequence Read Archive (SRA) accession numbers for the generated dataset. (*G. aculeatus and P. pungitus*, represent the three- and nine-spined stickleback, respectively).

Specimen	Library name	Species	Population	Number of PE ^a^reads	Source	SRA accession
Gas1	LibB3-PP	*G. aculeatus*	White sea	75,948,125	This study	SRR11611415
Gas2	Lib311	*G. aculeatus*	White sea	73,927,012	This study	SRR11611416
Pun1	LibB2-P9M ^b^	*P. pungitius*	White sea	124,576,543	This study	SRR11611426
Pun2	LibChu82	*P. pungitius*	White sea	129,103,432	This study	SRR11611420
Pun3	LibChu83	*P. pungitius*	White sea	81,714,662	This study	SRR11611421
Pun4	LibD1	*P. pungitius*	White sea	69,716,994	This study	SRR11611422
Pun5	LibChM42	*P. pungitius*	White sea	54,187,021	This study	SRR11611418
Pun6	LibChM72	*P. pungitius*	White sea	75,794,201	This study	SRR11611419
Pun7	LibB9-1	*P. pungitius*	White sea	70,767,251	This study	SRR11611417
Pun8	LibK3	*P. pungitius*	White sea	54,073,367	This study	SRR11611423
Pun9	LibK7	*P. pungitius*	White sea	58,358,776	This study	SRR11611424
GasJ	Japan3	*G. aculeatus*	Japan	104,697,312	Yoshida et al. (2019)	DRR067872
PunJ	Japan9	*P. pungitius*	Japan	142,279,188	Yoshida et al. (2019)	DRR013346

a PE, reads–paired-end reads.

b LibB2-P9M corresponds to SH3 specimen in Nedoluzhko et al. (2021).

**TABLE 3:** Genome mapping efficiency (ME) and breadth of coverage (BC) percentages (%) of the three fish species reference genomes.

Specimen name	Library name	Ref. genome 1		Ref. genome 2		Ref. genome 3	
		*G. aculeatus*		*P. pungitius*		*D. labrax*	
		ME	BC	ME	BC	ME	BC
Gas1	LibB3-PP	85.36	95.00	17.36	28.95	4.82	4.30
Gas2	Lib311	91.30	94.58	18.54	29.72	—	—
Pun1	LibB2-P9M	17.94	50.55	77.54	84.71	3.78	3.74
Pun2	LibChu82	9.84	30.16	42.64	84.58	2.14	4.68
Pun3	LibChu83	11.50	29.64	47.34	84.32	2.49	4.77
Pun4	LibD1	17.61	29.17	75.22	84.57	3.91	3.76
Pun5	LibChM42	20.53	25.70	74.64	82.09	5.76	4.02
Pun6	LibChM72	12.72	28.24	53.46	84.30	3.11	4.54
Pun7	LibB9-1	18.64	28.02	79.66	84.43	3.98	3.57
Pun8	LibK3	21.06	27.74	79.27	83.34	4.96	4.46
Pun9	LibK7	19.42	28.64	80.15	84.32	4.21	4.28
GasJ	Japan3	98.85	91.11	20.56	27.90	5.05	4.02
PunJ	Japan9	17.96	27.81	17.91	39.45	3.84	3.41

Note that all quantities are expressed as percentage (%).

### 3.1 Genotype analysis of stickleback specimens

**FIGURE 2:**
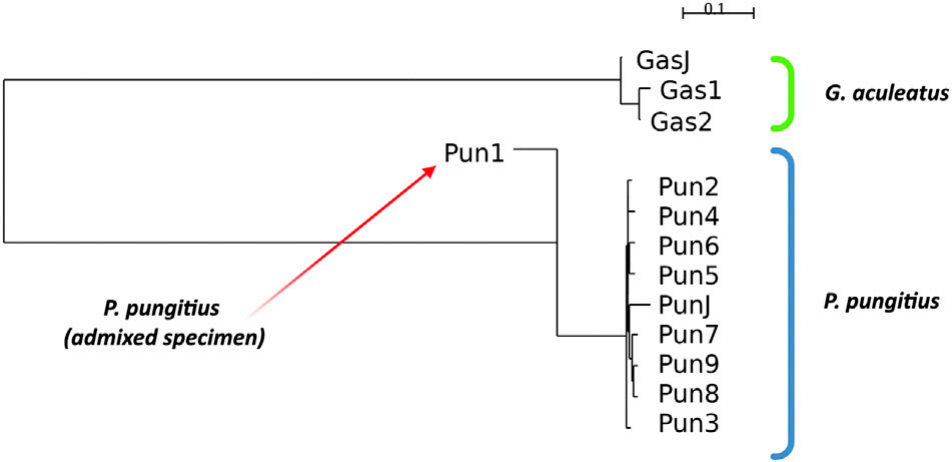
Neighbor-joining clustering of three- and nine-spined sticklebacks based on Nei’s distances. Admixed specimen of nine-spined stickleback species is indicated by a red arrow.

To perform all standard measurements for our specimens, we performed a cluster analysis of the specimens based on Nei’s distances. The results of neighbor-joining clustering are shown in Figure 2. The admixed specimen, which was described in a previous study (Nedoluzhko et al., 2021), clustered more distantly from other nine-spined stickleback specimens. Moreover, the branch of the admixed specimen in the neighbor-joining reconstructions is inverted against other specimens because this specimen has a negative Nei’s distance score. This phenomenon is obviously a result of incorrect operation of the neighbor-joining algorithm with non-bifurcation phylogenetic events, such as in the case of admixtures. When the algorithm tries to fit admixed data to a bifurcating tree, the clustering malfunctions and produces these inverted branches.

Other three- and nine-spined stickleback specimens show proper clustering according to their phylogenetic position. The neighbor-joining clustering reconstruction distinguishes the stickleback species quite well from each other, and specimens from Japanese populations are located in their species clusters among the White Sea stickleback population specimens.

### 3.2 Analysis of mobile elements

**FIGURE 3:**
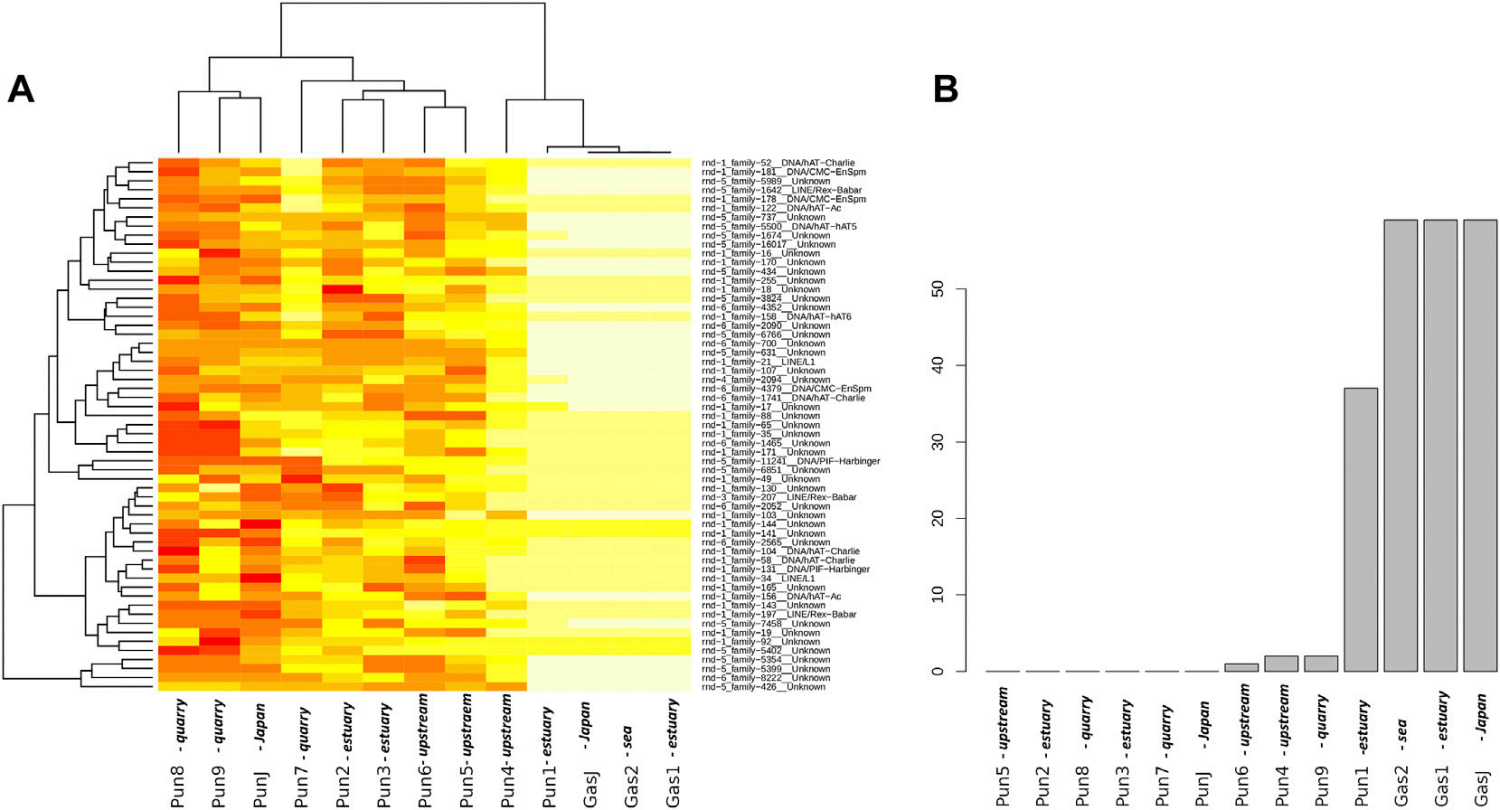
Analysis of transposable elements specific to the three-spined stickleback **(A)** Heatmap of transposable element (TE) representation. Columns of the heatmap correspond to specimens, whereas rows correspond to transposable element families present in specimens. The red color on the heatmap indicates low coverage of the corresponding transposable element, whereas the white color shows high coverage. **(B)** Numbers of “three-spined” TE families present in specimens from the White Sea populations. Only TEs that were fully covered by sequencing reads and had a high mapping depth were taken into account. The specimens were sorted according to the number of TE families present. White Sea stickleback populations are marked as quarry, upstream, estuary, and sea according to the sampling location.

To evaluate the effect of an admixture on the distribution of mobile elements, we estimated TE profiles for each specimen. In particular, we were interested in “three-spined stickleback” TE distribution in the genomes of nine-spined stickleback. The results for three-spined TE expansion are presented in Figure 3. Here, we show only the TEs that were present in the Japanese three-spined stickleback specimen but were not present in the Japanese nine-spined stickleback specimen. We are thereby able to observe TE expansion from the genomes of three-spined stickleback into those of the nine-spined stickleback in the White Sea.

In total, we defined 442 TE families from the three-spined stickleback genome which were longer than 300 nucleotides in length. At the same time, only 216 TE families remained after filtering by depth of coverage (>100×). Such deep coverage is necessary to be sure of selecting the differences between nine-spined stickleback and three-spined stickleback TE sequences. We selected only TEs which were fully covered in length by sequences from Japanese three-spined stickleback specimens (DRR067872) but were not covered by Japanese nine-spined stickleback specimens (DRR013346). Finally, only 59 TEs remained, and all of them were present in three-spined White Sea three-spined stickleback specimens, as well as in Japanese specimens. We showed that the admixed nine-spined stickleback specimen (Pun1) significantly differed from other nine-spined stickleback specimens in terms of the number of “three-spined” transposable elements. It had 37 of 59 “three-spined” TEs, whereas most of the other nine-spined stickleback specimens had no such TE in its entirety (except Pun4, Pun6, and Pun9, which had only one or two fully covered TEs) (Figure 3B).

In this part of the study, we deliberately used Japanese three- and nine-spined stickleback SRA datasets while supposing that other nine-spined stickleback specimens from the White Sea population (in addition to the admixed Pun1 specimen) could also have traces of being admixing with three-spined stickleback. Our results allow us to assert that TE analyses are very sensitive to hybridization detection, obviously due to the ability of mobile elements to uncontrollably propagate in hybrid genomes.

### 3.3 ABBA-BABA test of “nine-spined” specimens

In our previous study using RAD-Seq with eight specimens of three- and nine-spined stickleback (Nedoluzhko et al., 2021), we defined traces of three-spined stickleback introgression into the genome of a single nine-spined stickleback specimen (Pun1).

**FIGURE 4:**
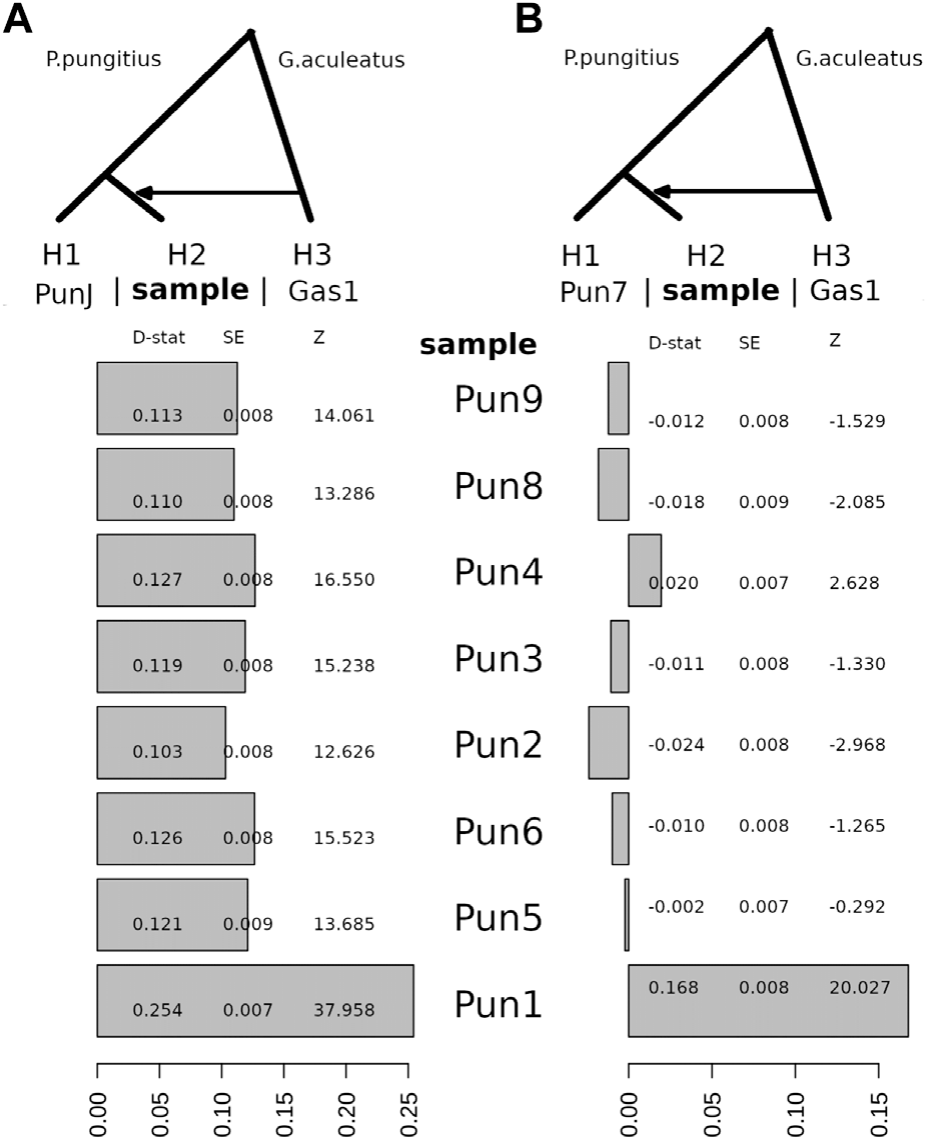
The D-statistic test indicates introgression between the White Sea three-spined stickleback, *G. aculeatus* specimen (Gas1) and all of the White Sea nine-spined stickleback *P. pungitius* specimens with respect to the Japanese nine-spined stickleback specimen **(A)** and the White Sea nine-spined stickleback specimen **(B)**. Compared with the Japanese nine-spined stickleback, all of the White Sea nine-spined stickleback specimens have considerable introgression levels from three-spined stickleback. D-stat, the result of D-statistic test; SE, standard error; Z, z-score.

Here, using the WGS data, we decided to repeat this type of analysis for 11 three-spined stickleback and nine-spined stickleback specimens in order to obtain more precise results. To demonstrate the possibility of admixture between these two species, we used an ABBA-BABA test, among other methods. Surprisingly, although we estimated a three-spined stickleback introgression level when comparing a nine-spined stickleback specimen from the Japanese population (DRR013346), all White Sea nine-spined stickleback specimens as well as the admixed specimen (Pun1) had traces of admixing with three-spined stickleback (Figure 4A). Positive D-statistic value indicates an allelic shift between H2 to H3 specimens, compared to H1 specimen.

Compared with the Japanese nine-stickleback specimen, all tested nine-spined stickleback specimens from the White Sea had traces of admixture with three-spined stickleback in their genomes; moreover, the statistical support for this admixture is quite robust (Figure 4B). It is well-known that results with Z-scores greater than three in absolute value qualify as being statistically significant (Green et al., 2010). Here, we definitively showed that the Z-score for the White Sea nine-spined stickleback freshwater population varies from 12.6 to 37.9. This evidence suggests that a background level of hybridization exists between the three- and nine-spined stickleback inhabiting the White Sea basin.

It should also be noted that when we use the nine-spined stickleback reference (Pun7) to test the admixture level compared with other White sea specimens, we found that only the Pun1 specimen, which was previously found, showed a high Z-score. The admixture level of other specimens is hidden because the Pun7 specimen has the same level of admixture by itself.

### 3.4 Distribution of introgressed loci

**FIGURE 5:**
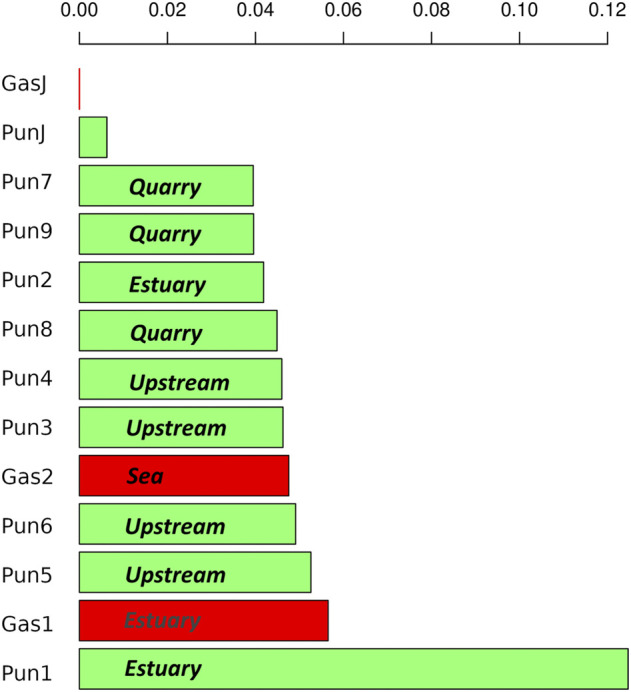
Bar chart of normalized number of reads mapped to the “alien” genome. Three-spined stickleback specimens marked in red; nine-spined stickleback specimens marked in green. White Sea stickleback population marked as quarry, upstream, estuary, and sea, according to the sampling location.

To estimate the admixture level for White Sea nine-spined stickleback specimens, we counted DNA reads which mapped to the reference genome of three-spined stickleback but not that of nine-spined stickleback. We showed that the Pun1 specimen, previously described as admixed, had the greatest number of “three-spined stickleback” reads. At the same time, other White Sea nine-spined stickleback specimens also host high numbers of these “alien” reads. Moreover, White Sea three-spined stickleback specimens had a much greater number of “nine-spined” reads than Japanese specimens (Figure 5). The Japanese nine-spined stickleback specimen’s mapping efficiency was lower than that inhabiting the White Sea (Table 3). Most probably, this difference contributed to the lower number of alien origin reads in this specimen.

The Japanese nine-spined stickleback specimen’s mapping efficiency was lower than that inhabiting the White Sea (Table 3). Most probably, this difference contributed to the lower number of alien origin reads in this specimen.

Regarding the methods, on a general line, while the alien read analysis provides evidence on the number of genome segments of alien origin incorporated to the derivatives of the white-sea nine-spined stickleback, the D-statistic test is supported on the SNP alleles.

Moreover, for conserved genome regions of three- and nine-spined stickleback genomes, the alien read analysis show the possibility of introgression, while the D-statistic test did not support this because genotypes in this locus are the same. In contrast, alien reads analysis was more efficient in analyzing the loci of transposable or repetitive elements (TEs) due to their high density in the vertebrate genomes and probability of bias during genotype analysis.

The assessment of introgressed loci in the source (three-spined stickleback) and target genome (nine-spined stickleback) revealed their nonuniform distribution across the genome. Using tests and correlation procedures described by Favorov and colleagues (Favorov et al., 2012), we estimated the distribution of source introgressed loci in three-spined stickleback genome versus genes and versus transposable element locations (Table 4).

**TABLE 4:** Analysis of relative distances and intersections of introgressed loci with protein-coding genes and transposable elements (TE) versus source of introgressed loci in three-spined stickleback genome and target introgressed loci for LibB2-B9M nine-spined stickleback genome is presented.

	Source of introgression		Target of introgression	
Statistic name	Introgressed loci vs. TE	Introgressed loci vs. genes	Introgressed loci vs. TE	Introgressed loci vs. genes
Query population (fragments)	123,104	120,716	157,417	29,013
Reference population (fragments)	494,288	22,270	303,483	7,405
Query coverage (nt.)	15,945,795	15,603,801	365,694,254	21,596,797
Reference coverage (nt.)	66,062,554	189,993,805	95,089,834	28,967,551
Relative distances Kolmogorov-Smirnov *p*-value	0	0.0001518632	0.0498455334	0.0020492999
Relative distances ECDF deviation area	0.02075064	0.001288394	0.0006375816	0.0033102375
Relative distances ECDF area correlation	0.08293058	−0.00525788	0.0015108528	0.0130496244

Briefly, the ECDF test evaluates whether two sets of genomic intervals are spatially correlated across the entire genome. The deviation from the uneven distribution of one set of intervals in relation to another is estimated. The indicator of unevenness is relative distances empirical cumulative distribution function (ECDF) deviation area, while if the value is greater than zero, then the correlations between the two intervals are positive, and if less than zero, then the correlations are negative. If the test value is zero, then the distribution of the second interval, relative to the first, is random. The ECDF correlation value for TE-containing intervals was positive, but was negative for gene intervals, with positive values coinciding with the occurrence of introgressed loci with mobile elements. Our results reveal the tendency of introgressed alleles to coincide with transposable elements, but not in three-spined stickleback genes (Table 4). Table 4 shows the distribution of introgression fragments for an admixed specimen (Pun1) and contains the following information—the quantity of genomic intervals of each type (query/reference.population), the total length of the intervals in nucleotides (query/reference.coverage), and the relative distance statistics from each genomic interval of the query set to the nearest interval of the reference set. Low *p*-values suggest a high significance of the non-uniformity in introgressed locus distribution across genomes.

### 3.5 Gene ontology analysis of introgressed loci

Previously described “alien” DNA reads (Figure 5) were used for the *de novo* assembly of “alien” DNA contigs. A BLAST search allowed us to identify their orthologs in the three-spined stickleback reference genome. We showed that several of these contigs were aligned to protein-coding sequences, despite the fact that the majority of introgressed loci tended to be located in repeat and transposable element regions (Table 4). We defined the genes which corresponded to introgressed sequences in an admixed specimen (Pun1), performed the functional annotation of the genes, and reveal the gene categories most enriched for the list of introgressed genes (Table 5).

**TABLE 5:** Gene ontology (GO) analysis of introgressed stickleback genes, conducted using the Database for Annotation, Visualization and Integrated Discovery (DAVID v6.8) for functional annotation by gene ontology (GO) categories. This analysis assigned 205 genes, revealed by BLAST searching the database of three-spined stickleback genes for *de novo* assembly of “alien” contigs. The results are limited using a false discovery rate (FDR) value of 1%.

Category	Term	Number of counts	%	*p-value*	Pop hits	Fold enrichment	FDR
UP_KEYWORDS	Alternative splicing	150	73.2	1.97E-10	10587	1.422	2.54E-07
UP_KEYWORDS	Membrane	112	54.6	1.09E-07	7494	1.5	1.40E-04
UP_SEQ_FEATURE	Glycosylation site: N-linked (e.g., GlcNAc)	77	37.6	5.09E-08	4234	1.797	7.97E-05
UP_KEYWORDS	Glycoprotein	77	37.6	5.93E-07	4551	1.699	7.64E-04
UP_KEYWORDS	Cell membrane	64	31.2	1.93E-08	3175	2.024	2.49E-05
UP_SEQ_FEATURE	Topological domain: cytoplasmic	64	31.2	7.69E-07	3456	1.83	1.20E-03
UP_SEQ_FEATURE	Topological domain: extracellular	54	26.3	2.37E-06	2787	1.915	3.70E-03
UP_KEYWORDS	Cell adhesion	27	13.2	2.03E-12	479	5.659	2.62E-09
UP_KEYWORDS	Calcium	25	12.2	6.86E-06	877	2.862	8.83E-03
UP_KEYWORDS	Cell junction	24	11.8	2.70E-07	675	3.57	3.48E-04
GOTERM_BP_DIRECT	Homophilic cell adhesion *via* plasma membrane adhesion molecules	12	5.8	1.94E-06	158	6.642	3.08E-03
GOTERM_CC_DIRECT	GO:0043204, perikaryon	10	4.9	1.97E-06	106	8.817	2.56E-03
UP_SEQ_FEATURE	Domain: cadherin 2	10	4.9	2.49E-06	115	8.594	3.90E-03
UP_SEQ_FEATURE	Domain: cadherin 1	10	4.9	2.49E-06	115	8.594	3.90E-03
INTERPRO	Cadherin	10	4.9	5.32E-06	118	7.825	7.65E-03
INTERPRO	Cadherin-like	10	4.9	6.10E-06	120	7.694	8.78E-03
UP_SEQ_FEATURE	Domain: EGF-like 2	9	4.4	3.23E-06	89	9.994	5.06E-03
UP_SEQ_FEATURE	Repeat: desmoglein repeat 2	4	1.9	3.99E-06	4	98.833	6.25E-03
UP_SEQ_FEATURE	Repeat: desmoglein repeat 1	4	1.9	3.99E-06	4	98.833	6.25E-03
INTERPRO	Desmoglein	4	1.9	4.89E-06	4	92.333	7.04E-03

## 4 Discussion

Interspecific hybridization between closely related species is a very common event in nature. It allows species to improve their ecological potential, increase genetic diversity, and conquer new habitats (Lee, 2002; Chiba, 2005; Dlugosch and Parker, 2008; Prentis et al., 2008; Pereira et al., 2014a; Nichols et al., 2015; Yoshida et al., 2016). In teleost genomes, traces of such hybridization are widely presented in different taxa (Pereira et al., 2014b; Dowling et al., 2016; Yamasaki et al., 2020). Intergeneric hybridization in natural conditions is a rare process; previously, it was described only in few cases, including fish species (LeClere et al., 2012; Dowling et al., 2016).

The *Gasterosteus* and *Pungitius* species (predominantly three- and nine-spined stickleback) are well-known scientific models which became popular in evolutionary and ecological studies decades ago (Ziuganov et al., 1987; Merila, 2013; Reid et al., 2021). Recently published studies have described the possibility of successful hybridization among *Pungitius* species (*P. sinensis*, *P. tymensis*, *P. platygaster*, and *P. pungitius*) (Kobayashi, 1959; Yamasaki et al., 2020) as well as among *Gasterosteus* species (*G. aculeatus* and *G. nipponicus*) (Yoshida et al., 2016). Moreover, it has been shown that two species, three- and nine-spined stickleback, can have fertile intergeneric artificial hybrids (Hart, 2003). The three- and the nine-spined stickleback are eurybiont species, which are ecologically similar in many aspects (including taste preferences); they also have the same chromosome numbers and sympatric distribution in the Northern hemisphere (Mikhailova and Kasumyan, 2006; Tibblin et al., 2020). Our previous study showed the possibility of occasional intergeneric hybridization between three- and nine-spined stickleback inhabiting northwestern Russia (Nedoluzhko et al., 2021).

The main result of this study is the discovery that the exchange of genetic material between distant taxa is much more common than previously expected. The modern biological species concept assumes a species as a group of organisms that are genetically isolated from other species (Butlin and Stankowski, 2020). Based on the biological species concept interspecific hybridization, as mentioned above, occurs only between evolutionary close species and usually stops on F1 generation hybrids because of their infertility or low fertility. But in the study, we describe hybridization traces in genomes of fairly distant species belonging to different genera. Intriguing that all nine-spined stickleback specimens studied undergoing intergeneric introgression. Our findings are unexpected and surprising since they contradicted the view of the interspecific barrier and seem to suggest a possible important biological role of introgression.

In the present study, using whole-genome sequencing of three- and nine-spined stickleback specimens from the White Sea basin, we clearly show the presence of a moderate level of introgression between three- and nine-spined stickleback species distributed in this location. By contrast, the Japanese nine-spined stickleback specimen did not have a significant shift in allele frequencies compared with three-spined stickleback*.* We suggest that the moderate level of allele shift in the nine-spined stickleback population and the relatively low genetic distance between White Sea nine-spined stickleback and three-spined stickleback specimens are related to admixture events between these two species in this region of moderate salinity. Our results also show that the introgression process between the two species happens as a regular event across the White Sea specimens and populations included in this study. This is indicated by the results of D-statistic tests of three-spined stickleback specimens versus White Sea nine-spined stickleback as well as versus Japanese nine-spined stickleback specimens. If we assume the adaptive nature of introgression associated with adaptation to marine conditions, then the presence of introgression in freshwater specimens can be explained by irregular interbreeding with marine individuals who migrate from time to time between marine and freshwater populations. Whether this is an adaptive genomic introgression or a consequence of accidental hybridization of these species remains unclear and will be analyzed in subsequent studies.

We also show that genotype analysis of specimens that have introgression events during evolution could have bias because most clustering tools assume a divergence sequence model and are unable to adequately handle admixed data (Balaban et al., 2019). Taking into account the high prevalence of interspecific hybridization in natural populations (Mallet, 2005), prior to clustering, it would probably be reasonable to determine and exclude admixed specimens from these types of analyses.

TE analysis has a dramatic ability to define introgression traces in specimens that have relatively recent admixture events (Belyayev, 2014). Despite the D-statistic test determining a moderate level of admixture in all White Sea specimens, TE analysis defined traces of introgression only in the Pun1 specimen. However, we found a very strong sign of admixture in this nine-spined stickleback specimen, whereby it had 62.7% of the most abundant “three-spined” TEs. Theoretically, interspecific transposon transfer can occur through parasites (Piskurek and Jackson, 2012). Nevertheless, distinct loci with interspecific origin were observed in the White Sea nine-spined stickleback genomes, in addition to transposable elements. Thus, an assumption about hybridization between these two stickleback species makes more sense.

Considering the number of “aliens” reads described in this study, we revealed a higher introgression rate in the White Sea nine-spined stickleback population compared with the Japanese specimen. This finding can indicate different interspecies relationships in sticklebacks across the Northern hemisphere. It was recently shown that the Japanese three-spined stickleback hybridizes with the closely related species *G. nipponicus* (Yoshida et al., 2016) in their native habitats. Our data demonstrate that in the moderate-salinity White Sea basin, three-spined stickleback found a more evolutionary distant mating partner.

Analysis of the proximity of introgressed loci of admixed specimens to protein-coding gene and transposable element positions showed a high number of intersections with three-spined stickleback TEs. ECDF correlation analysis revealed a negative correlation value for introgressed loci versus protein-coding genes and the converse with TE loci. This means that the sources of the introgressed loci tend to be located in transposable element sequences. Nevertheless, intragenic sequences were less common among them than would be expected due to randomness. We suppose that the introgression to protein-coding sequences could lead to conflict with other genes (peptides) of the hybrid, as was suggested for PSI (Dobzhansky, 1940), whereas mobile elements could spread across the genome more freely without obstacles. Moreover, it is also important to note that transposable elements play a significant role in microevolutionary processes in populations, and the exchange of these elements between species should have evolutionary significance (Bonchev and Parisod, 2013).

Interestingly, analysis of the targets of introgressed loci in the nine-spined stickleback genome showed that they are more likely to be located in gene-rich regions (Table 5), whereas the ECDF area correlation between introgression targets and TEs is close to zero and statistical support for this is also low (slightly less than 5%). This supports the assumption that the loci of the admixed specimen (Pun1), in which the genetic material of the three-spined stickleback was introgressed, are randomly distributed regarding transposons, but are “attracted” to genes. This implies that introgression has functional significance and possibly affects gene expression in the outbred organisms.

Gene ontology analysis of loci that introgressed to the nine-stickleback genome revealed categories associated with cell membranes. The cell membrane is a compartment that acts as the barrier of the cell and is more exposed to the environment (Lombard, 2014). It is possible that loci introgressed from three-spined stickleback result in three-spined stickleback × nine-spined stickleback hybrids being more resistant to brackish water.

The content of introgressed genes associated with alternative gene splicing was also observed to be increased in an admixed specimen (Pun1). We speculate that this could be a mechanism of adaptation to genome disturbance, which is usually associated with hybridization. As we have already pointed out, according to the PSI hypothesis, parent allele incompatibility appears in hybrid genomes. The linkage between introgression and alternative splicing has previously been shown to be a rather common phenomenon, even in archaic humans (Rotival et al., 2019). We suppose that alternative splicing loci can increase allele variability, thereby reducing hybrid incompatibility.

In the present study, a direct association of the introgression process mediated by the White Sea’s moderate salinity is not revealed. However, we speculate that this unique environmental condition under specific conditions occasionally controls the hybridization event between three- and nine-spined sticklebacks. Furthermore, the genomic analysis showed that such events remain rare but allow an increase in the genetic diversity of local populations and are possibly an alternative option in the adaptation processes to survive the global climate and environmental changes.

## Data Availability

The datasets presented in this study can be found in online repositories. The names of the repository/repositories and accession number(s) can be found below: www.ncbi.nlm.nih.gov/, PRJNA529064.

## References

[B1] Abbott R. Albach D. Ansell S. Arntzen J. W. Baird S. J. Bierne N. (2013). Hybridization and speciation. J. Evol. Biol. 26, 229–246. 10.1111/j.1420-9101.2012.02599.x 23323997

[B2] Arkhipova I. R. (2017). Using bioinformatic and phylogenetic approaches to classify transposable elements and understand their complex evolutionary histories. Mob. DNA-Uk 8, 19. 10.1186/s13100-017-0103-2 PMC5718144 29225705

[B3] Artemov A. V. Mugue N. S. Rastorguev S. M. Zhenilo S. Mazur A. M. Tsygankova S. V. (2017). Genome-Wide DNA methylation profiling reveals epigenetic adaptation of stickleback to marine and freshwater conditions. Mol. Biol. Evol. 34, 2203–2213. 10.1093/molbev/msx156 28873953

[B4] Balaban M. Moshiri N. Mai U. Jia X. F. Mirarab S. (2019). TreeCluster: Clustering biological sequences using phylogenetic trees. Plos One 14, e0221068. 10.1371/journal.pone.0221068 31437182 PMC6705769

[B5] Bankevich A. Nurk S. Antipov D. Gurevich A. A. Dvorkin M. Kulikov A. S. (2012). SPAdes: A new genome assembly algorithm and its applications to single-cell sequencing. J. Comput. Biol. 19, 455–477. 10.1089/cmb.2012.0021 22506599 PMC3342519

[B6] Belyayev A. (2014). Bursts of transposable elements as an evolutionary driving force. J. Evol. Biol. 27, 2573–2584. 10.1111/jeb.12513 25290698

[B7] Bonchev G. Parisod C. (2013). Transposable elements and microevolutionary changes in natural populations. Mol. Ecol. Resour. 13, 765–775. 10.1111/1755-0998.12133 23795753

[B8] Borkin L. J. Litvinchuk S. N. (2013). Animal hybridization, speciation and systematics. Tr. Zool. Instituta RAN 2, 83–139.

[B9] Butlin R. K. Stankowski S. (2020). Is it time to abandon the biological species concept? No. Natl. Sci. Rev. 7, 1400–1401. 10.1093/nsr/nwaa109 34692168 PMC8288981

[B10] Camacho C. Coulouris G. Avagyan V. Ma N. Papadopoulos J. Bealer K. (2009). BLAST+: Architecture and applications. BMC Bioinforma. 10, 421. 10.1186/1471-2105-10-421 PMC2803857 20003500

[B11] Chiba S. (2005). Appearance of morphological novelty in a hybrid zone between two species of land snail. Evol. 59, 1712–1720. 10.1554/04-521.1 16329242

[B12] Cui R. Schumer M. Kruesi K. Walter R. Andolfatto P. Rosenthal G. G. (2013). Phylogenomics reveals extensive reticulate evolution in Xiphophorus fishes. Evolution 67, 2166–2179. 10.1111/evo.12099 23888843

[B13] Dlugosch K. M. Parker I. M. (2008). Founding events in species invasions: Genetic variation, adaptive evolution, and the role of multiple introductions. Mol. Ecol. 17, 431–449. 10.1111/j.1365-294X.2007.03538.x 17908213

[B14] Dobzhansky T. (1940). Speciation as a stage in evolutionary divergence. Am. Nat. 74, 312–321. 10.1086/280899

[B15] Dowling T. E. Markle D. F. Tranah G. J. Carson E. W. Wagman D. W. May B. P. (2016). Introgressive hybridization and the evolution of lake-adapted catostomid fishes. PLoS One 11, e0149884. 10.1371/journal.pone.0149884 26959681 PMC4784955

[B16] Favorov A. Mularoni L. Cope L. M. Medvedeva Y. Mironov A. A. Makeev V. J. (2012). Exploring massive, genome scale datasets with the GenometriCorr package. PLoS Comput. Biol. 8, e1002529. 10.1371/journal.pcbi.1002529 22693437 PMC3364938

[B17] Flutre T. Duprat E. Feuillet C. Quesneville H. (2011). Considering transposable element diversification in de novo annotation approaches. PLoS One 6, e16526. 10.1371/journal.pone.0016526 21304975 PMC3031573

[B18] Ford A. G. Dasmahapatra K. K. Ruber L. Gharbi K. Cezard T. Day J. J. (2015). High levels of interspecific gene flow in an endemic cichlid fish adaptive radiation from an extreme lake environment. Mol. Ecol. 24, 3421–3440. 10.1111/mec.13247 25997156 PMC4973668

[B19] Green R. E. Krause J. Briggs A. W. Maricic T. Stenzel U. Kircher M. (2010). A draft sequence of the Neandertal genome. Science 328, 710–722. 10.1126/science.1188021 20448178 PMC5100745

[B20] Hart P. J. B. (2003). Habitat use and feeding behaviour in two closely related fish species, the three-spined and nine-spined stickleback: An experimental analysis. J. Anim. Ecol. 72, 777–783. 10.1046/j.1365-2656.2003.00747.x

[B21] Howe K. L. Contreras-Moreira B. De Silva N. Maslen G. Akanni W. Allen J. (2020). Ensembl Genomes 2020-enabling non-vertebrate genomic research. Nucleic Acids Res. 48, D689–D695. 10.1093/nar/gkz890 31598706 PMC6943047

[B22] Huang D. W. Sherman B. T. Lempicki R. A. (2009). Systematic and integrative analysis of large gene lists using DAVID bioinformatics resources. Nat. Protoc. 4, 44–57. 10.1038/nprot.2008.211 19131956

[B23] Jombart T. Ahmed I. (2011). Adegenet 1.3-1: new tools for the analysis of genome-wide SNP data. Bioinformatics 27, 3070–3071. 10.1093/bioinformatics/btr521 21926124 PMC3198581

[B24] Jones F. C. Grabherr M. G. Chan Y. F. Russell P. Mauceli E. Johnson J. (2012). The genomic basis of adaptive evolution in threespine sticklebacks. Nature 484, 55–61. 10.1038/nature10944 22481358 PMC3322419

[B25] Kinsella R. J. Kahari A. Haider S. Zamora J. Proctor G. Spudich G. (2011). Ensembl BioMarts: A hub for data retrieval across taxonomic space. Database (Oxford) 2011, bar030. 10.1093/database/bar030 21785142 PMC3170168

[B26] Knaus B. J. Grunwald N. J. (2017). vcfr: a package to manipulate and visualize variant call format data in R. Mol. Ecol. Resour. 17, 44–53. 10.1111/1755-0998.12549 27401132

[B27] Kobayashi H. (1959). Cross-experiments with three species of stickleback, Pungitius pungitius (L.), Pungitius tymensis (Nikolsk), and Pungitius sinensis (Guichenot), with special reference to their systematic relationship. J. Hokkaido Gakugei Univ. Sect. B 10, 363–384.

[B28] Korneliussen T. S. Albrechtsen A. Nielsen R. (2014). Angsd: Analysis of next generation sequencing data. BMC Bioinforma. 15, 356. 10.1186/s12859-014-0356-4 PMC4248462 25420514

[B29] Langdon Q. K. Peris D. Kyle B. Hittinger C. T. sppIDer (2018). sppIDer: A species identification tool to investigate hybrid genomes with high-throughput sequencing. Mol. Biol. Evol. 35, 2835–2849. 10.1093/molbev/msy166 30184140 PMC6231485

[B30] Langmead B. Salzberg S. L. (2012). Fast gapped-read alignment with Bowtie 2. Nat. Methods 9, 357–359. 10.1038/nmeth.1923 22388286 PMC3322381

[B31] Lawrence M. Huber W. Pages H. Aboyoun P. Carlson M. Gentleman R. (2013). Software for computing and annotating genomic ranges. PLoS Comput. Biol. 9, e1003118. 10.1371/journal.pcbi.1003118 23950696 PMC3738458

[B32] LeClere J. B. Hoaglund E. P. Scharosch J. Smith C. E. Gamble T. (2012). Two naturally occurring intergeneric hybrid snakes (*Pituophis catenifer* × *Pantherophis vulpinus*; lampropeltini, squamata) from the midwestern United States. J. Herpetology 46, 257–262. 10.1670/10-260

[B33] Lee C. E. (2002). Evolutionary genetics of invasive species. Trends Ecol. Evol. (Amst.) 17, 386–391. 10.1016/s0169-5347(02)02554-5

[B34] Li H. Handsaker B. Wysoker A. Fennell T. Ruan J. Homer N. (2009). The sequence alignment/map format and SAMtools. Bioinformatics 25, 2078–2079. 10.1093/bioinformatics/btp352 19505943 PMC2723002

[B35] Lombard J. (2014). Once upon a time the cell membranes: 175 years of cell boundary research. Biol. Direct 9, 32. 10.1186/s13062-014-0032-7 25522740 PMC4304622

[B36] Lucek K. Roy D. Bezault E. Sivasundar A. Seehausen O. (2010). Hybridization between distant lineages increases adaptive variation during a biological invasion: Stickleback in Switzerland. Mol. Ecol. 19, 3995–4011. 10.1111/j.1365-294X.2010.04781.x 20735738

[B37] Mallet J. Besansky N. Hahn M. W. (2016). How reticulated are species? Bioessays 38, 140–149. 10.1002/bies.201500149 26709836 PMC4813508

[B38] Mallet J. (2005). Hybridization as an invasion of the genome. Trends Ecol. Evol. 20, 229–237. 10.1016/j.tree.2005.02.010 16701374

[B39] Merila J. (2013). Nine-spined stickleback (*Pungitius pungitius*): An emerging model for evolutionary biology research. Ann. N. Y. Acad. Sci. 1289, 18–35. 10.1111/nyas.12089 23550583

[B40] Mikhailova E. S. Kasumyan A. O. (2006). Comparison of taste preferences in the three-spined *Gasterosteus aculeatus* and nine-spined *Pungitius pungitius* sticklebacks from the White sea basin. J. Ichthyol. 46, S151–S160. 10.1134/s003294520611004x

[B41] Nedoluzhko A. Sharko F. Tsygankova S. Boulygina E. Ibragimova A. Teslyuk A. (2021). Genomic evidence supports the introgression between two sympatric stickleback species inhabiting the White Sea basin. Heliyon 7, e06160. 10.1016/j.heliyon.2021.e06160 33604473 PMC7875830

[B42] Nichols P. Genner M. J. van Oosterhout C. Smith A. Parsons P. Sungani H. (2015). Secondary contact seeds phenotypic novelty in cichlid fishes. Proc. Biol. Sci. 282, 20142272. 10.1098/rspb.2014.2272 25392475 PMC4262179

[B43] Paradis E. Schliep K. (2019). Ape 5.0: an environment for modern phylogenetics and evolutionary analyses in R. Bioinformatics 35, 526–528. 10.1093/bioinformatics/bty633 30016406

[B44] Payseur B. A. Rieseberg L. H. (2016). A genomic perspective on hybridization and speciation. Mol. Ecol. 25, 2337–2360. 10.1111/mec.13557 26836441 PMC4915564

[B45] Pembleton L. W. Cogan N. O. Forster J. W. (2013). StAMPP: an R package for calculation of genetic differentiation and structure of mixed-ploidy level populations. Mol. Ecol. Resour. 13, 946–952. 10.1111/1755-0998.12129 23738873

[B46] Pereira C. S. A. Aboim M. A. Rab P. Collares-Pereira M. J. (2014). Introgressive hybridization as a promoter of genome reshuffling in natural homoploid fish hybrids (Cyprinidae, Leuciscinae). Heredity 112, 343–350. 10.1038/hdy.2013.110 24220087 PMC3931169

[B47] Pereira R. J. Barreto F. S. Burton R. S. (2014). Ecological novelty by hybridization: Experimental evidence for increased thermal tolerance by transgressive segregation in *Tigriopus californicus* . Evolution 68, 204–215. 10.1111/evo.12254 24372605

[B48] Piskurek O. Jackson D. J. (2012). Transposable elements: From DNA parasites to architects of metazoan evolution. Genes. (Basel) 3, 409–422. 10.3390/genes3030409 24704977 PMC3899998

[B49] Prentis P. J. Wilson J. R. Dormontt E. E. Richardson D. M. Lowe A. J. (2008). Adaptive evolution in invasive species. Trends Plant Sci. 13, 288–294. 10.1016/j.tplants.2008.03.004 18467157

[B50] Quinlan A. R. Hall I. M. (2010). BEDTools: A flexible suite of utilities for comparing genomic features. Bioinformatics 26, 841–842. 10.1093/bioinformatics/btq033 20110278 PMC2832824

[B51] Rastorguev S. M. Nedoluzhko A. V. Gruzdeva N. M. Boulygina E. S. Tsygankova S. V. Oshchepkov D. Y. (2018). Gene expression in the three-spined stickleback (*Gasterosteus aculeatus*) of marine and freshwater ecotypes. Acta Naturae 10, 66–74. 10.32607/20758251-2018-10-1-66-74 29713520 PMC5916735

[B52] Rastorguev S. M. Nedoluzhko A. V. Sharko F. S. Boulygina E. S. Sokolov A. S. Gruzdeva N. M. (2016). Identification of novel microRNA genes in freshwater and marine ecotypes of the three-spined stickleback (*Gasterosteus aculeatus*). Mol. Ecol. Resour. 16, 1491–1498. 10.1111/1755-0998.12545 27238497

[B53] Reid K. Bell M. A. Veeramah K. R. (2021). Threespine stickleback: A model system for evolutionary genomics. Annu. Rev. Genomics Hum. Genet. 22, 357–383. 10.1146/annurev-genom-111720-081402 33909459 PMC8415275

[B54] Rotival M. Quach H. Quintana-Murci L. (2019). Defining the genetic and evolutionary architecture of alternative splicing in response to infection. Nat. Commun. 10, 1671. 10.1038/s41467-019-09689-7 30975994 PMC6459842

[B55] Rudman S. M. Schluter D. (2016). Ecological impacts of reverse speciation in threespine stickleback. Curr. Biol. 26, 490–495. 10.1016/j.cub.2016.01.004 26804556

[B56] Runemark A. Vallejo-Marin M. Meier J. I. (2019). Eukaryote hybrid genomes. PLoS Genet. 15, e1008404. 10.1371/journal.pgen.1008404 31774811 PMC6880984

[B57] Sambrook J. Russell D. W. Sambrook J. (2006). The condensed protocols from molecular cloning : A laboratory manual. Cold Spring Harbor, N.Y.: Cold Spring Harbor Laboratory Press.

[B58] Sharko F. S. Nedoluzhko A. V. Le B. M. Tsygankova S. V. Boulygina E. S. Rastorguev S. M. (2019). A partial genome assembly of the miniature parasitoid wasp, *Megaphragma amalphitanum* . Plos One 14, e0226485. 10.1371/journal.pone.0226485 31869362 PMC6927652

[B59] Souissi A. Bonhomme F. Manchado M. Bahri-Sfar L. Gagnaire P. A. (2018). Genomic and geographic footprints of differential introgression between two divergent fish species (*Solea* spp.). Heredity 121, 579–593. 10.1038/s41437-018-0079-9 29713088 PMC6221876

[B60] Taylor E. B. Boughman J. W. Groenenboom M. Sniatynski M. Schluter D. Gow J. L. (2006). Speciation in reverse: Morphological and genetic evidence of the collapse of a three-spined stickleback (*Gasterosteus aculeatus*) species pair. Mol. Ecol. 15, 343–355. 10.1111/j.1365-294X.2005.02794.x 16448405

[B61] Taylor S. A. Larson E. L. (2019). Insights from genomes into the evolutionary importance and prevalence of hybridization in nature. Nat. Ecol. Evol. 3, 170–177. 10.1038/s41559-018-0777-y 30697003

[B62] Terekhanova N. V. Barmintseva A. E. Kondrashov A. S. Bazykin G. A. Mugue N. S. (2019). Architecture of parallel adaptation in ten lacustrine threespine stickleback populations from the White sea area. Genome Biol. Evol. 11, 2605–2618. 10.1093/gbe/evz175 31406984 PMC6761963

[B63] Terekhanova N. V. Logacheva M. D. Penin A. A. Neretina T. V. Barmintseva A. E. Bazykin G. A. (2014). Fast evolution from precast bricks: Genomics of young freshwater populations of threespine stickleback *Gasterosteus aculeatus* . PLoS Genet. 10, e1004696. 10.1371/journal.pgen.1004696 25299485 PMC4191950

[B64] Tibblin P. Hall M. Svensson P. A. Merila J. Forsman A. (2020). Phenotypic flexibility in background-mediated color change in sticklebacks. Behav. Ecol. 31, 950–959. 10.1093/beheco/araa041 32760177 PMC7390996

[B65] Urton J. R. McCann S. R. Peichel C. L. (2011). Karyotype differentiation between two stickleback species (gasterosteidae). Cytogenet. Genome Res. 135, 150–159. 10.1159/000331232 21921583 PMC3224509

[B66] Varadharajan S. Rastas P. Loytynoja A. Matschiner M. Calboli F. C. F. Guo B. (2019). A high-quality assembly of the nine-spined stickleback (*Pungitius pungitius*) genome. Genome Biol. Evol. 11, 3291–3308. 10.1093/gbe/evz240 31687752 PMC7145574

[B67] Wallis G. P. Cameron-Christie S. R. Kennedy H. L. Palmer G. Sanders T. R. Winter D. J. (2017). Interspecific hybridization causes long-term phylogenetic discordance between nuclear and mitochondrial genomes in freshwater fishes. Mol. Ecol. 26, 3116–3127. 10.1111/mec.14096 28295830

[B68] Yamasaki Y. Y. Kakioka R. Takahashi H. Toyoda A. Nagano A. J. Machida Y. (2020). Genome-wide patterns of divergence and introgression after secondary contact between *Pungitius* sticklebacks. Philos. Trans. R. Soc. Lond. B Biol. Sci. 375, 20190548. 10.1098/rstb.2019.0548 32654635 PMC7423276

[B69] Yoshida K. Ishikawa A. Toyoda A. Shigenobu S. Fujiyama A. Kitano J. (2019). Functional divergence of a heterochromatin-binding protein during stickleback speciation. Mol. Ecol. 28, 1563–1578. 10.1111/mec.14841 30117211

[B70] Yoshida K. Miyagi R. Mori S. Takahashi A. Makino T. Toyoda A. (2016). Whole-genome sequencing reveals small genomic regions of introgression in an introduced crater lake population of threespine stickleback. Ecol. Evol. 6, 2190–2204. 10.1002/ece3.2047 27069575 PMC4782248

[B71] Ziuganov V. V. Golovatjuk G. J. Savvaitova K. A. Bugaev V. F. (1987). Genetically isolated sympatric forms of threespine stickleback, *Gasterosteus aculeatus*, in lake azabachije (Kamchatka-Peninsula, ussr). Environ. Biol. Fishes 18, 241–247. 10.1007/bf00004877

