# Supplementary material for: Intergeneric hybridization of two stickleback species leads to introgression of membrane-associated genes and invasive TE expansion
Source: Front Genet. 2022 Aug 25;13:863547. doi: 10.3389/fgene.2022.863547 (PMC9452749; doi:10.3389/fgene.2022.863547)
Supplement: Supplementary file 1 [file DataSheet1.PDF]

Supplementary for:

Intergeneric Hybridization of Two Stickleback Species Leads to Introgression of Membrane-Associated Genes and Invasive TE Expansion

Artem Nedoluzhko [1], Fedor Sharko [2,3,4], Svetlana Tsygankova [5], Eugenia Boulygina [5], Natalia Slobodova [5], Anton Teslyuk [6], Jorge Galindo-Villegas [7*] and Sergey Rastorguev [2,3,4*]

[1]Paleogenomics laboratory, European University at Saint Petersburg, Saint Petersburg, Russia

[2]Laboratory of Vertebrate Genomics and Epigenomics, Research Center of Biotechnology of the Russian Academy of Sciences, Moscow, Russia

[3]Limited liability company ELGENE, Moscow, Russia.
[4]Laboratory of bioinformatics and big data analysis, Kurchatov Center for Genomic Research, National Research Centre "Kurchatov Institute", Moscow, Russia
[5]Laboratory of Eukaryotic Genomics, Kurchatov Center for Genomic Research, National Research Centre "Kurchatov Institute", Moscow, Russia
[6]National Research Centre "Kurchatov Institute", Moscow, Russia
[7]Faculty of Biosciences and Aquaculture, Nord University, Bodø, Norway

*** Correspondence:**
Jorge Galindo-Villegas
jorge.galindo-villegas@nord.no

Sergey Rastorguev
rastorgueff@gmail.com

Supplementary Table 1. Genome mapping efficiency (ME) and breadth of coverage (BC) percentages (%) of the three fish species reference genomes.

**Specimen names**	**Ref. genome 1** ***G. aculeatus***		**Ref. genome 2** ***P. pungitius***		**Ref. genome 3** ***D. labrax***	
	ME	BC	ME	BC	ME	BC
Japan3	98.85	91.11	20.56	27.90	5.05	4.02
Japan9	17.96	27.81	17.91	39.45	3.84	3.41
Lib311	91.30	94.58	18.54	29.72	-	-
LibB2-P9M 2	17.94	50.55	77.54	84.71	3.78	3.74
LibB3-PP	85.36	95.00	17.36	28.95	4.82	4.30
LibB9-1	18.64	28.02	79.66	84.43	3.98	3.57
LibChM42	20.53	25.70	74.64	82.09	5.76	4.02
LibChM72	12.72	28.24	53.46	84.30	3.11	4.54
LibChu82	9.84	30.16	42.64	84.58	2.14	4.68
LibChu83	11.50	29.64	47.34	84.32	2.49	4.77
LibD1	17.61	29.17	75.22	84.57	3.91	3.76
LibK3	21.06	27.74	79.27	83.34	4.96	4.46
LibK7	19.42	28.64	80.15	84.32	4.21	4.28

Note that all quantities are expressed as percentage (%).
